# Prognostic value of *PDL1* expression in pancreatic cancer

**DOI:** 10.18632/oncotarget.11685

**Published:** 2016-08-29

**Authors:** David J. Birnbaum, Pascal Finetti, Alexia Lopresti, Marine Gilabert, Flora Poizat, Olivier Turrini, Jean-Luc Raoul, Jean-Robert Delpero, Vincent Moutardier, Daniel Birnbaum, Emilie Mamessier, François Bertucci

**Affiliations:** ^1^ Département d'Oncologie Moléculaire, Centre de Recherche en Cancérologie de Marseille, INSERM, CNRS, Université Aix-Marseille, Marseille, France; ^2^ Département de Chirurgie Générale et Viscérale, AP-HM, Marseille, France; ^3^ Faculté de Médecine, Aix-Marseille Université, Marseille, France; ^4^ Equipe de Médecine Translationelle Hépato-gastro-entérologie, Institut Paoli-Calmettes, Marseille, France; ^5^ Département d'Oncologie Médicale, Institut Paoli-Calmettes, Marseille, France; ^6^ Département d'Oncologie Chirurgicale, Institut Paoli-Calmettes, Marseille, France; ^7^ Département d'Anatomopathologie, Institut Paoli-Calmettes, Marseille, France

**Keywords:** expression, immune response, pancreatic cancer, PDL1, survival

## Abstract

Pancreatic cancer is one of the most aggressive human cancers. PD1/PDL1-inhibitors recently showed promising results in different cancers with correlation between PDL1 tumor expression and responses. Expression of programmed cell death receptor ligand 1 (PDL1) has been scarcely studied in pancreatic cancer. In this retrospective study, we analyzed *PDL1* mRNA expression in 453 clinical pancreatic cancer samples profiled using DNA microarrays and RNASeq. Compared to normal pancreatic samples, *PDL1* expression was upregulated in 19% of cancer samples. Upregulation was not associated with clinicopathological features such as patients' age and sex, pathological type, tumor size, lymph node status, and grade, but was associated with shorter disease-free survival and overall survival in multivariate analyses. Analysis of correlations with biological parameters showed that *PDL1* upregulation was associated with some degree of lymphocyte infiltration and signs of anti-tumor T-cell response, but to a lesser extent than what has been reported in breast cancer and GIST. PDL1-up pancreatic cancers displayed profiles of lymphocyte exhaustion, were more enriched in inhibitory molecules and pro-tumor populations (Tregs with upregulation of *FOXP3* and *IL10*, myeloid-derived suppressor cells with upregulation of *CD33* and *S100A8/A9*), and demonstrated a down-modulation of most MHC class I members (*HLA-A/B/C*, *HLA-E/F/G*) suggestive of a defect in antigen processing and presentation. In conclusion, our results suggest that *PDL1* expression might refine the prediction of metastatic relapse in operated pancreatic cancer, and that PD1/PDL1 inhibitors might reactivate inhibited T-cells to increase the anti-tumor immune response in PDL1-upregulated tumors.

## INTRODUCTION

Pancreatic ductal adenocarcinoma is a major public health problem worldwide with 260,000 deaths annually [1] and its incidence is rising [2]. Early radical resection of the tumor is the only potentially curative treatment, but at diagnosis less than 20% of patients are eligible for surgery. The inoperability and the poor prognosis are due to late diagnosis, propensity to rapid dissemination to lymph nodes and distant organs (>80% of patients displayed metastases at diagnosis) [3], early recurrences after resection, and poor response to available systemic therapies [4, 5]. The median survival in patients with inoperable or metastatic pancreatic cancer is of 6 months from diagnosis and the long-term survival is null. During the past 20 years, research efforts have mainly focused on improvements in radiotherapy and systemic treatments. Today, there are only few chemotherapeutic agents that have shown to be effective against pancreatic carcinoma, including gemcitabine with or without nab-paclitaxel as well as the FOLFIRINOX regimen that combines 5-FU, leucovorin, oxaliplatin and irinotecan. Unfortunately, the survival benefit remains modest, making crucial the development of novel drugs. The success of immunotherapy in other cancers and various evidences for the role of immunity in pancreatic carcinoma [6–11] have suggested that immunotherapy can be a promising alternative in pancreatic cancer. One strategy is the development of immune checkpoint inhibitors that are changing the current treatment paradigm for cancers.

Immune response is balanced between activator and inhibitor pathways that regulate the activity of tumor-infiltrating lymphocytes (TILs). This balance may be disturbed in cancers, where the inhibition of the immune system favors tumor progression. The PD1 pathway plays a major role in the negative regulation of cell-mediated immune responses. PD1 (Programmed cell Death 1) is expressed at the surface of various immune cells including T-cells. PD1 is activated by its ligands, PDL1 or PDL2, expressed by antigen-presenting cells such as macrophages or B-cells but also by cancer cells. The PDL1-PD1 interaction attenuates lymphocyte activation [12–16], promotes regulatory T-cell development and function, and impairs anti-tumor T-cell immune response. PD1 or PDL1 inhibitors have shown very promising results in clinical trials notably in melanoma and renal, lung, prostate and bladder carcinomas [17–19], with durable tumor responses or stabilizations. In some cases, a relationship has been reported between therapeutic response and PDL1 expression on tumor and/or immune cells [17, 18, 20–22].

PDL1 expression has been described in different cancers such as breast, kidney, lung, esophagus, ovary, colorectal, head and neck and squamous cell carcinomas, melanomas, GIST and gliomas [23–36]. In pancreatic cancer, PDL1 expression has been very scarcely studied [27, 37–41], with the largest study analyzing 81 cases using immunohistochemistry (IHC). Here, we have analyzed *PDL1* mRNA expression in 453 clinical pancreatic cancer samples profiled using DNA microarrays and RNASeq. We searched for correlations between *PDL1* expression and clinico-pathological data, including survival.

## RESULTS

### PDL1 expression and clinicopathological features

We analyzed *PDL1* mRNA expression in 453 clinical pancreatic cancer samples pooled from nine data sets. Their clinicopathological characteristics are summarized in Supplementary Table 1. Ninety-nine percent of cases were ductal carcinoma and all but one had been initially treated by surgery. *PDL1* expression was variable among the 453 samples with a wide range of values over 3 decades in log_2_ scale, suggesting heterogeneous expression across samples (Supplementary Figure 1). We searched for correlations between *PDL1* mRNA expression assessed as binary variable and available clinicopathological features. We thus defined two groups of cancer samples based upon *PDL1* expression in tumors compared with mean expression in normal pancreatic samples: the “*PDL1*-up” group (*N* = 87; 19%) and the “*PDL1*-not-up” group (*N* = 366, 81%). As shown in Table 1, the *PDL1* groups were not associated with patient's age and sex, and pathological features such as pathological type, tumor size, lymph node status and tumor grade.

**Table 1:** *PDL1* expression and clinicopathological features

Characteristics		PDL1 expression groups			
		*N*	not-up (*N* = 366)	up (*N* = 87)	*p*
Median age, years (range)		207	65 (32-88)	64.5 (44-84)	0.929
Sex					0.336
	female	128	92 (44%)	36 (51%)	
	male	154	119 (56%)	35 (49%)	
Pathological tumor size (pT)					0.113
	1	8	6 (4%)	2 (4%)	
	2	32	29 (19%)	3 (6%)	
	3	161	117 (76%)	44 (88%)	
	4	3	2 (1%)	1 (2%)	
Pathological lymph node status (pN)					0.292
	negative	62	50 (33%)	12 (24%)	
	positive	139	102 (67%)	37 (76%)	
Pathological type					1.000
	ductal	422	341 (93%)	81 (93%)	
	other	31	25 (7%)	6 (7%)	
Pathological tumor grade					0.511
	1	33	26 (17%)	7 (14%)	
	2	102	79 (53%)	23 (47%)	
	3	63	44 (30%)	19 (39%)	
Surgery					1.000
	no	1	1 (0%)	0 (0%)	
	yes	452	365 (100%)	87 (100%)	
Bailey's molecular subtypes					1.00E-04
	ADEX*	109	95 (26%)	14 (16%)	
	immunogenic	69	53 (14%)	16 (18%)	
	pancreatic progenitor	101	93 (25%)	8 (9%)	
	squamous	174	125 (34%)	49 (56%)	
Median DFS follow-up, months (range)		254	9.86 (1-84)	1.30 (1-24)	1.77E-05
2-year DFS		254	37% [0.3-0.46]	14% [0.07-0.31]	3.19E-04
Median OS follow-up, months (range)		254	9.53 (1-84)	2.99 (1-31)	4.14E-05
2-year OS		254	50% [0.42-0.6]	22% [0.11-0.42]	7.06E-05

* ADEX, aberrantly differentiated endocrine exocrine

Finally, a recent study published a transcriptional classification of pancreatic ductal carcinoma identifying four tumor subtypes (squamous, pancreatic progenitor; immunogenic; and aberrantly differentiated endocrine exocrine) with different molecular pathways and prognosis [42]. We applied this classification to our dataset and found a significant correlation with the PDL1 status: 56% of PDL1-up tumors were in the squamous subtype (Table 1), the one with the worst prognosis. This was confirmed by the fact that PDL1 mRNA was up-regulated in the squamous subtype vs other subtypes in the Bailey's data set [42] and in our own data set (data not shown).

### PDL1 expression and survival

**Figure 1:**
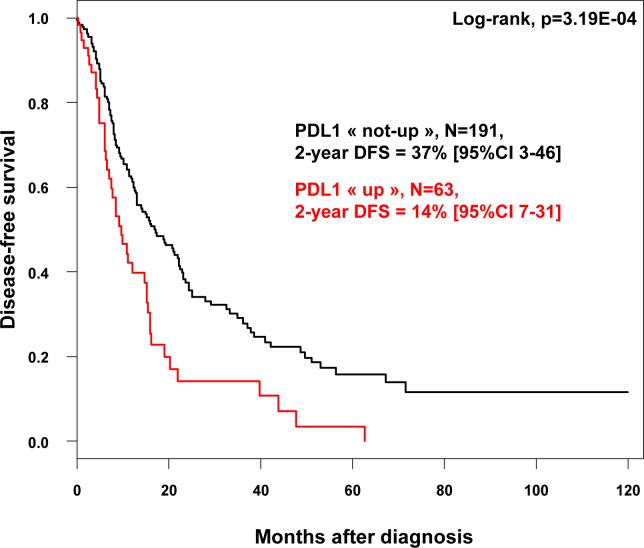
Disease-free survival according to PDL1 mRNA expression in patients with pancreatic cancer Kaplan-Meier DFS curves in patients with high and low expression in the whole population.

We assessed the prognostic value of *PDL1* expression in terms of disease-free survival (DFS) and overall survival (OS). DFS and OS data were available for 254 patients non-metastatic at diagnosis and treated with surgery. Regarding DFS, the median follow-up was 7.5 months (range, 1-84) months, 163 patients displayed a DFS event, and the 2-year DFS was 32% (95%CI, 26-40). As shown in Figure 1, *PDL1* expression influenced DFS with 14% 2-year DFS (95%CI, 7-31) in the “PDL1-up” group *versus* 37% (95%CI, 3-46) in the “PDL1-not-up” group (*p* = 0.00032, log-rank test). The HR for DFS event was 1.90 (95%CI, 1.33-2.70) in the “PDL1-up” group *versus* “PDL1-not-up” group (*p* = 0.0004, Wald test). The median DFS was 6 months (range, 1 to 63) in “PDL1-up” group *versus* 10.7 months (range, 1 to 156) in “PDL1-not-up” group. In univariate analysis (Table 2), *PDL1* expression, large pathological tumor size, lymph node involvement, pathological type, and high tumor grade were associated with DFS, whereas age and sex were not. In multivariate analysis, *PDL1* expression remained the sole prognostic feature for DFS (Table 2).

**Table 2:** Univariate and multivariate Cox regression analyses for DFS

Characteristics		Univariate			Multivariate		
		N	HR [95CI]	p	N	HR [95CI]	p
Age		182	1.02 [1-1.03]	0.0785	174	1.02 [1-1.03]	0.091
Sex	male vs. female	212	1.2 [0.79-1.57]	0.529			
pT	2 vs. 1	180	0.93 [0.26-3.4]	4.06E−02	174	0.51 [0.13-1.95]	0.322
	3 vs. 1		2.02 [0.63-6.4]		174	0.64 [0.18-2.25]	0.490
pN	pos vs. neg	177	1.83 [1.16-2.88]	8.94E-03	174	1.49 [0.9-2.48]	0.125
Type	other vs. ductal	254	0.45 [0.24-0.83]	1.12E-02	174	0.84 [0.41-1.71]	0.630
Grade	2 vs. 1	180	1.70 [0.92-3.15]	1.40E-02	174	1.21 [0.64-2.3]	0.551
	3 vs. 1		2.46 [1.31-4.61]		174	1.58 [0.83-3.01]	0.166
*PDL1* group	up vs. not-up	254	1.9 [1.33-2.7]	4.04E-04	174	1.75 [1.12-2.74]	1.49E-02

**Figure 2:**
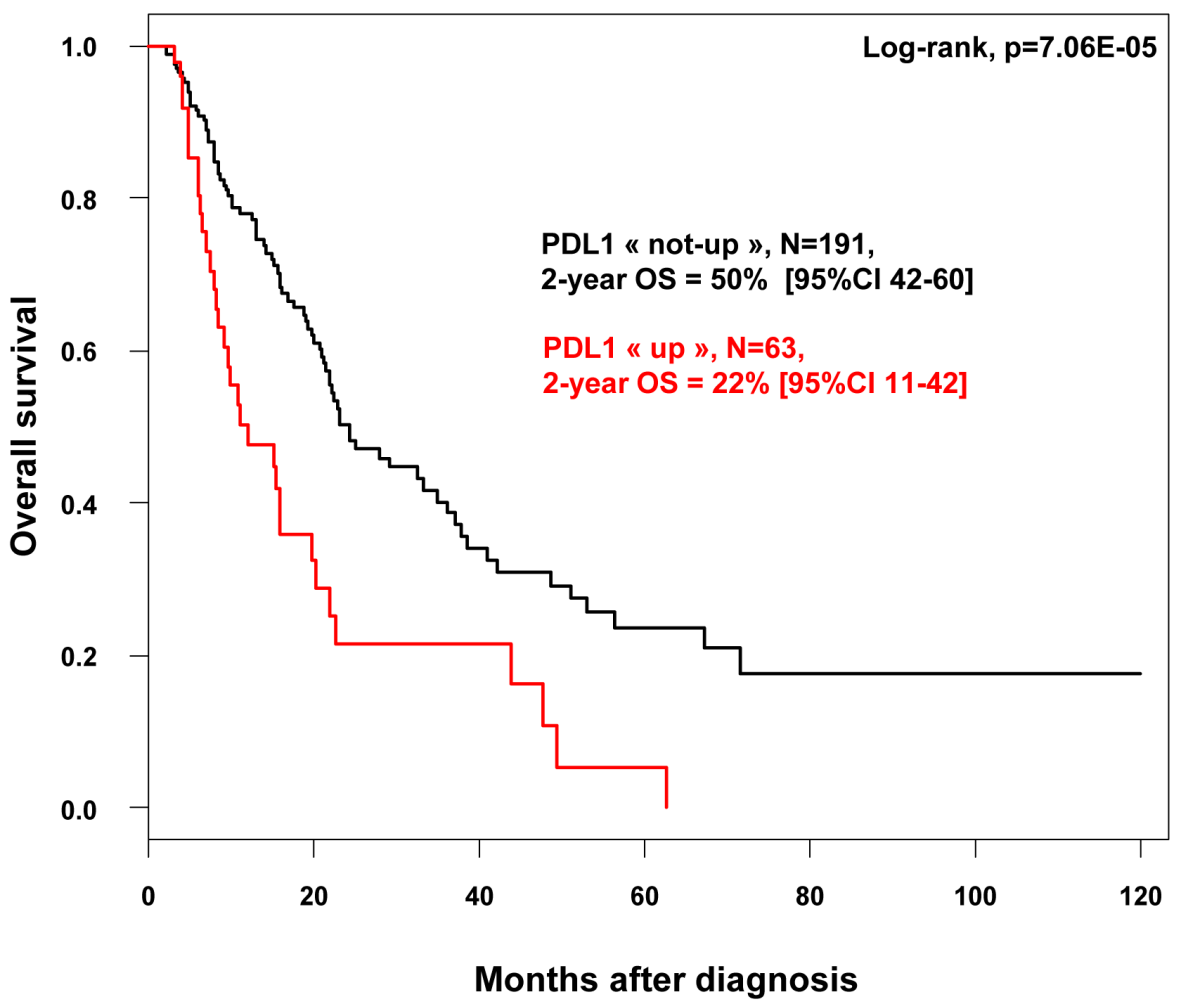
Overall survival according to PDL1 mRNA expression in patients with pancreatic cancer Kaplan-Meier OS curves in patients with high and low expression in the whole population.

Similar results were observed for OS. With a median follow-up of 7.5 months (range, 1-84) months, 120 patients died, and the 2-year OS was 44% (95%CI, 37-53). As shown in Figure 2, the 2-year OS rate was 50% (95%CI, 42-60) in the “PDL1-not-up” group *versus* 22% (95%CI, 11-42] in the “PDL1-up” group (*p* = 7.06E-05, log-rank test). The median OS was 6.4 months (range, 1 to 63) in “PDL1-up” group *versus* 11.4 months (range, 1 to 156) in “PDL1-not-up” group. In univariate analysis (Table 3), *PDL1* expression, age, large pathological tumor size, lymph node involvement, pathological type, and high tumor grade, were associated with poor OS, whereas sex was not. The HR for death was 2.22 (95%CI, 1.48-3.33) in the “PDL1-up” group *versus* “PDL1-not-up” group (*p* = 0.0001, Wald test). In multivariate analysis, *PDL1* expression and age remained the sole prognostic features for OS (Table 3). Of note, PDL1 expression remains an independent prognostic factor for DFS and OS in multivariate analysis including the Bailey's molecular classification (data not shown).

**Table 3:** Univariate and multivariate Cox regression analyses for OS

Characteristics		Univariate			Multivariate		
		N	HR [95CI]	p	N	HR [95CI]	p
Age		182	1.03 [1-1.05]	1.86E-02	174	1.03 [1-1.06]	1.87E-02
Sex	male vs. female	212	1.05 [0.69-1.59]	0.818			
pT	2 vs. 1	180	1.31 [0.15-11.3]	2.74E-02	174	0.6 [0.06-6.12]	0.663
	3 vs. 1		3.98 [0.55-28.9]		174	0.84 [0.05-13.44]	0.899
pN	pos vs. neg	177	2.02 [1.11-3.69]	2.14E-02	174	1.4 [0.68-2.88]	0.365
Type	other vs. ductal	254	0.15 [0.05-0.47]	1.15E-03	174	0.59 [0.17-2.04]	0.407
Grade	2 vs. 1	180	2.27 [0.93-5.50]	1.81E-02	174	1.47 [0.59-3.67]	0.410
	3 vs. 1		3.45 [1.42-8.42]		174	1.85 [0.74-4.6]	0.185
*PDL1* group	up vs. not-up	254	2.22 [1.48-3.33]	1.08E-04	174	2.36 [1.34-4.16]	2.96E-03

### PDL1 expression and associated biological processes

Supervised analysis applied to the largest data set (TCGA set, *N* = 178) identified 2,405 genes differentially expressed between the tumors with (*N* = 46) *versus* without (*N* = 132) *PDL1* upregulation, including 1,624 genes upregulated and 781 genes downregulated in the “PDL1-up” samples (Supplementary Figure 2A-2B; Supplementary Table 2). The robustness of this gene signature was confirmed in the pool of all other independent sets including a total of 275 tumors by using a metagene-based prediction score: as shown in Supplementary Figure 2C, the score was higher in the “PDL1-up” samples than in the “PDL1-not-up” samples (*p* = 2.0E-14, Student t-test). Ontology analysis of these 2,405 genes (Supplementary Table 3) revealed that “PDL1-up” tumors overexpressed genes involved in the regulation of the local immune response. More specifically, we found that numerous upregulated genes are involved in immune response, notably in inflammatory response (mostly Toll-Like Receptors, molecules of the complement cascade, but also *CD163*, *CD302*, *IL1R1*, *IL6*, *IL8*, and molecules involved in the synthesis of leukotrienes, phospholipids and prostaglandins) and lymphocytes chemotaxis. Major actors of leukocyte activation (*CD2*, *CD3D*, *CD3E*, *CD3G*, *CD4*, *CD5*, *CD8A*, *CD8B*, *CD27*, *CD28*, *CD38*, *CD40LG*, *CD80*, *CD86*, *CD226*, *CD247*, *HLA-DOA, HLA-DRA*, *KLRK1*, *TNFSF4*, *TNFSF14*, *TNFRSF8*, *IL2RA*, *IL7R*, *IL11*, *IL12RB1*, *IL12RB2*, *IL21R*, *IL31RA*, *IFNG*, *IKZF1*, *JAK2*, *PIK3CG*, *EOMES*, *RORA*, *TBX21*…) were also positively correlated with *PDL1* expression, attesting of a strong recruitment and tumor infiltration by T-cells in the “PDL1-up” group. However, numerous genes related to negative lymphocyte regulation were also present (*BTLA*, *CTLA4*, *FOXP3*, *HAVCR2*, *HIF1A*, *IL10*, *IL1RAP*, *IDO1*, *KLRC1*, *LAG3*, *PDCD1LG2*, *VSIG4*, *SPN*, *SLA2*, *TGFBR2*, *TGFB2*, *TGFB3*, *TIGIT*…), notably all the hallmark molecules of exhausted T-cells (*BTLA*, *HAVCR2*, *LAG3*, *PD1*). Many genes upregulated in the “PDL1-not-up” group are involved in cell metabolism.

### PDL1 expression and immune response-related features

Given the role of PDL1 in immunity, we searched for correlations between the two *PDL1* expression-based groups and immunity-related features (Supplementary Table 4). No correlation was found with the percentage of tumor-infiltrating lymphocytes (TILs), which was available in the TCGA data set, as previously reported [27, 41]. We found a correlation with the three Palmer's B-cell, T-cell, and CD8+ T-cell gene expression signatures, the expression module of each signature being higher in the “PDL1-up” group than in the “PDL1-not-up” group. Out of four gene expression signatures tested and reflecting the cytotoxic T-cell response, only one, the “LCK signature”, was associated with the PDL1 groups, whereas the three other ones (“medullary breast cancer”, “28-kinase immune”, and “immune response”) were not. Finally, we found that the probability of activation of immune-related pathways such as IFNα and IFNγ was higher in the “PDL1-up” group. Altogether, these results suggested that PDL1 expression in pancreatic cancer is associated with some degree of lymphocyte infiltration and signs of anti-tumor T-cell response.

### Comparison of PDL1-associated immune response-related features in pancreatic cancer, breast cancer and GIST

Given the opposite prognostic value of PDL1 expression in pancreatic cancer (unfavorable value) when compared with breast cancer and GIST (favorable value) [23, 25], we compared the biological and immune features associated with PDL1 expression in these cancers. First, we observed that the correlations described in the previous section were much weaker (Supplementary Table 4) than those we had previously reported in breast cancers and GISTs [23, 25], where *PDL1* upregulation was associated with stronger cytotoxic T-cell responses. The strength of association between *PDL1* expression and the probability of activation of immune-related pathways (IFNα, IFNγ) was also much lower in pancreatic carcinomas than in breast cancers and GISTs (Supplementary Table 4). This would be in line with the highlighted exhausted lymphocyte profile observed in the pancreatic carcinoma *PDL1* signature. Second, we compared the three *PDL1* gene signatures (pancreas, breast, GIST) that we generated *de novo* from the 12,091 genes common to the three studies and using the same parameters of supervised analysis (moderated t-test, *p* < 5%, *q* < 25%, |FC|>1.5x). The comparison of signatures (pancreas 1587 genes, breast 941 genes, GIST 1432 genes) revealed interesting differences regarding immune features (Supplementary Table 5). For example, all major markers of exhausted T-cells (*BTLA*, *HAVCR2*, *LAG3*, *PD1*) were present and strongly differential in the pancreas signature only. *FOXP3,* the master transcription factor for regulatory T-cells (Tregs), and its effective cytokine *IL10*, were also upregulated in the “PDL1-up” group of pancreatic cancers. The genes overexpressed in the PDL1-up group of pancreatic cancers also included CD33, but not CD14, which is concordant with the presence of myeloid-derived suppressor cells (MDSCs). Components of the S100A8/A9 complex, inflammatory mediators of immune suppression by MDSCs, were also overexpressed in this group. Finally, many genes related to antigen processing and presentation of exogenous peptide antigen via MHC class-I (*B2M*, *CYBB*, *HLA-A, HLA-B, HLA-C, HLA-E, HLA-F, HLA-G*, *NCF2, NCF4, PSMB8, PSMB9, PSMB10, PSME1, TAP1, TAP2, TAPBP*) were present in the PDL1 breast and GIST signatures, but not in the pancreatic signature, notably on the aspects of TAP-dependent mechanisms of antigen presentation, endocytosis, phagosome maturation (*CYBB*, *LTF, NCF1, NCF2, NCF4, RAB31*) and proteasomal ubiquitin-independent protein catabolic process. That was clearly shown by the ontology analysis of genes upregulated in these three signatures (Supplementary Table 6). Altogether, these observations converged toward a poorer efficiency of anti-tumor response in pancreatic carcinoma than in breast cancer and GIST, which can be monitored through *PDL1* transcript evaluation.

## DISCUSSION

Overexpression of PDL1 by tumor cells has been noted in a number of human cancers, and the blockade of the PD1-PDL1 pathway is a promising therapeutic approach in oncology. PDL1 inhibitors produced anti-tumor responses in mouse models of pancreatic cancers [27, 41, 43]. In this study, we have analyzed the *PDL1* mRNA expression in 453 clinical pancreatic cancer samples: *PDL1* upregulation was observed in 19% of cases and was associated with shorter DFS and OS in multivariate analysis.

To date, PDL1 expression in cancers has been mainly studied at the protein level using IHC. However, such analysis on paraffin-embedded slides has been a challenge until recently, with different non-standardized techniques and scoring systems making the results not conclusive [44, 45]. Indeed, the measurement of PDL1 expression by IHC is not yet standardized and many discordant results have been reported across studies, notably regarding the prognostic value of PDL1 expression [46]. Several antibodies are available but lack specificity and reproducibility [45, 47, 48] and the optimal positivity cut-off is not defined [49]. Given our current state of knowledge, the use of different PDL1 IHC assays as a “companion diagnostic” still raises many issues. Efforts to clarify the optimal IHC assay are ongoing for improvements, in most solid tumors, and in particular in pancreatic cancer [50]. On the other hand, a positive relationship between protein and mRNA PDL1 expression (using in situ fluorescent RNAscope paired-primer assay or ISH) has been reported in breast carcinoma [51].

In the present retrospective study, we have based our analysis on mRNA expression measured using DNA microarrays or RNASeq. Such approach allowed us to avoid the limitations of IHC and to work on a very large series of samples. Furthermore, the whole-genome aspect provided opportunity for better understanding how this co-inhibitory signaling molecule might contribute to the suppression of antitumor immunity in the tumor's microenvironment. Indeed, personalized cancer immunotherapy should integrate in the future not only the evaluation of PDL1 expression but also specific mechanisms through which cancer adapts to evade an anti-tumor immune response. We believe that gene expression profiling might contribute to highlight some of the markers that should be analyzed in this prospect, together with PDL1. Further studies are required to identify the right biomarkers able to predict and monitor response to the different PDL1/PD1 blocking agents. This might lead to a more or less complex algorithm that pathologists and biologists should consider rapidly [52].

To our knowledge, only six studies in the literature have described *PDL1* expression in pancreatic carcinoma and correlations with tumor features [27, 37–41]. Four studies were based on IHC [27, 37, 40, 41] and included from 8 to 81 patients, and two were based on IHC and qRT-PCR [38, 39] and included 40 patients each. We found *PDL1* upregulation in 19% of samples, lower than the percentages reported in these smaller series (from 32 to 62%), likely because of different scoring systems and analytic levels. Analysis of correlations with clinicopathological features showed discordant results between all studies including ours: we and others [27, 39] did not find any correlation with patients' age and sex, nor with pathological tumor size, lymph node status and grade, whereas two studies reported correlations between PDL1 expression and higher stage and higher tumor grade [37, 38]. By contrast, the unfavorable prognostic value of *PDL1* expression we report here was found in all three previous small series [27, 37, 39]; importantly, the larger size of our series (254 non-metastatic cases informative for survival) allowed the confirmation of its impact in multivariate analysis. Finally, we found a correlation between the PDL1 status and a recent transcriptional classification of pancreatic ductal carcinoma [42]: 56% of PDL1-up tumors were in the squamous subtype, and PDL1 mRNA was up-regulated in the squamous subtype versus each other subtype (pancreatic progenitor; immunogenic; and aberrantly differentiated endocrine exocrine). Interestingly, PDL1 expression remains an independent prognostic factor in multivariate analysis including the Bailey's molecular subtypes. Considering the immunosuppressive function of PDL1, it was not surprising to find expression associated with poor survival, as already reported in other cancers [52–57]. However, PDL1 expression has also been associated with favorable outcome in certain cancers such as breast and lung cancers [23, 24, 46, 51] and GIST [25]. Such opposition regarding the prognostic value of PDL1 expression in different cancers led us to study and compare the association between *PDL1* expression and biological and immune features. We thus looked at the genes correlating with *PDL1* expression to provide a better biological characterization of *PDL1*-up pancreatic carcinomas. These genes were almost exclusively related to immune cells and attested of tumor infiltration by lymphocytes. The comparison between three *PDL1* signatures revealed a stronger cytotoxic profile correlated with anti-tumor pathways activation (IFNα and IFNγ) in breast cancers and GISTs, suggesting a sustained activation of anti-tumor T-cells in breast cancers and GISTs of good prognosis. In these cases, we hypothesize that *PDL1* was upregulated because of a negative feedback loop that follows cytotoxic cells activation, notably through the production of IFNG, a, a known regulator of PDL1 expression. In the pancreatic cancer study, we also found IFNG expression as being correlated with *PDL1* expression, as reported by others [39]. However, this increased expression of IFNG was also associated to several elements that were not or were only partially present in the breast and GIST *PDL1* signatures, and that might provide some explanatory hints. First, all the typical markers related to T-cell exhaustion were upregulated in the “PDL1-up” pancreas group, in addition to *IDO1* and *CTLA4,* known as major actors attenuating T-cell immune response, in parallel to a major hypoxic environment (HIF1A). Second, *FOXP3,* the master transcription factor for Tregs, and its effective cytokine IL10, were associated with *PDL1* upregulation in pancreatic cancers. Interleukin-10 has strong immunosuppressive effects on lymphocyte activation, notably T_H_1 cytotoxic cells. In agreement with our data, Geng *et al* reported an increased prevalence of tumor-infiltrating Tregs in PDL1-positive pancreatic carcinomas [38, 39]. In parallel, IL-10 has been associated with poor survival in pancreatic cancer [39]. One explanation might be that *PDL1* stimulates IL-10 production [58]. Activation of Tregs, via IL-10 production, might thus represent a mechanism of downregulating the antitumor response through *PDL1*-upregulation. Third, we found upregulation of the *CD33* transcript, but not of *CD14*, suggesting the presence of myeloid-derived suppressor cells (MDSCs), previously reported as increased in pancreatic cancer [59]. MDSCs suppress T-cell responses in cancer patients and animal models [59]. In agreement with their likely infiltration, we found upregulation of the S100A8/A9 complex, an inflammatory mediator of immune suppression by MDSCs in PDL1-upregulated pancreatic cancers. This specific complex was previously involved in *PDL1* upregulation [60]. Finally, when looking at genes present in the breast and GIST *PDL1*-up signatures, but absent from the pancreas signature, we found many genes related to antigen presentation, including endocytosis and proteasome processing, highlighting a defect in antigen processing and presentation to anti-tumor immunity, notably to T-cells, in pancreatic carcinoma. Indeed, cytotoxic T-cells recognize, via their T-cell receptors (TCRs) and CD8 co-receptors, small antigenic peptides presented by the major histocompatibility complex (pMHC) of class I on the surface of all nucleated cells, including malignant cells. Our signature clearly demonstrated a down-modulation of most MHC class I members (HLA-A/B/C, HLA-E/F/G). This is a classical mechanism of escape in various malignant cells. It has been widely speculated that IL-10 could favor the development of tumors through immuno-suppressive mechanisms, including the modulation of antigen-presenting cells and especially dendritic cells functions [61, 62]. Altogether, these differences suggested that *PDL1*-up pancreatic cancers were more enriched in inhibitory molecules and pro-tumoral populations (Treg, MDSC), than *PDL1*-up breast cancers and GIST. Among the incriminated actors, some (IFNG, IL10, MDSC…) are directly implicated in and might explain the upregulation of PDL1 in pancreatic cancer. Altogether, this might explain the more pronounced escape to anti-tumor immunity, and the negative prognostic value of *PDL1* transcript in this cancer.

In conclusion, we showed that *PDL1* mRNA expression, observed in 19% of cases, represents an independent poor-prognosis feature for DFS and OS in pancreatic cancer. The main strength of our study lies in the number of samples analyzed (more than 450) and the parallel biological analyses. Limitations include the retrospective nature and associated biases, including the absence of information with respect to survival for all samples, and the use of gene expression profiling that quantifies expression level of both epithelial and stromal cells. Analysis of larger series, retrospective, then prospective is needed, as well as protein analysis when reliable antibodies are available. If confirmed, *PDL1* expression might refine the prognostication of operable pancreatic cancer and improve our ability to better tailor adjuvant therapy. From a therapeutic point of view, *PDL1* expression might guide the use of PD1/PDL1 inhibitors that could reactivate inhibited T-cells to increase the anti-tumor immune response when associated with primers of T-cell response such as immunogenic chemotherapy [63] or vaccines [41, 64]. Functional and clinical validation of this hypothesis is urgently warranted.

## MATERIALS AND METHODS

### Gene expression data sets

We gathered clinicopathological and gene expression data of clinical pancreatic carcinoma samples from nine publicly available data sets [65–72] (https://tcga-data.nci.nih.gov/tcga/) comprising at least one probe set representing *PDL1*. Data were collected from the National Center for Biotechnology Information (NCBI)/Genbank GEO, ArrayExpress, and TCGA databases. The nine data sets are described in Supplementary Table 7. Samples were profiled using whole-genome DNA microarrays (Affymetrix, Agilent, or Illumina) and RNASeq (Illumina). The pooled data set contained 565 samples, including 453 primary cancer samples and 112 normal pancreatic samples. The study was approved by our institutional board.

### Gene expression data analysis

Data analysis required pre-analytic processing. First, web http://ftp.ncbi.nlm.nih.gov/gene/ *PDL1* (*CD274*) tumour expression was measured by analysing different probe sets whose identity and specificity were verified using the NCBI program BLASTN 2.2.31+ (Supplementary Table 8). _2_We performed principal component analysis (PCA) using the top 2,000 most variable genes extracted from the nine data sets, before and after normalization, to verify the accuracy of the normalization in removing the set-specific variation in gene expression (Supplementary Figure 3). *PDL1* expression, before and after normalization, is shown for each dataset in Supplementary Figure 4. *PDL1*expression in tumors (T) was measured as discrete value after comparison with mean expression in the 112 normal pancreatic samples (NP): upregulation, thereafter designated “up” was defined by a T/NP ratio ≥2 and no upregulation (“not-up”) by a T/NP ratio < 2.

Because of the involvement of PDL1 in immunity, we analyzed gene expression signatures linked to immune response in cancers. Each of the following signatures was B-cell, T-cell, and CD8+ T-cell signatures [73], four signatures reflecting the cytotoxic T-cell response including the “LCK signature” [74], the “medullary breast cancer” signature [75], the “28-kinase immune” signature [76], and the “immune response” signature [77], and two Gatza's signatures of IFNα and IFNγ biological pathway activity [78]. Finally, to explore more-in-depth the biological pathways linked to *PDL1* expression in pancreatic cancer, we applied a supervised analysis by using the largest data set (TCGA: 178 samples) as learning set, and the other data sets as independent validation sets (275 samples). In the learning set, we compared the expression profiles of 20,531 genes between tumors with (*N* = 46) *versus* without (*N* = 132) *PDL1* upregulation using a moderated t-test with empirical Bayes statistic [79] included in the limma R packages. False discovery rate (FDR) [80] was applied to correct the multiple testing hypothesis and significant genes were defined by the following thresholds: *p* < 5%, *q* < 25% and fold change (FC) superior to |1.5x|. Ontology analysis of the resulting gene list was based on GO biological processes and Biocarta ontologies of the Database for Annotation, Visualization and Integrated Discovery (DAVID; david.abcc.ncifcrf.gov/). We verified the robustness of the resulting gene list in each validation set (a total of 41 tumors with and 234 without *PDL1* upregulation were represented) separately by computing for each tumor a metagene-based prediction score defined by the difference between the “metagene PDL1-up” (mean expression of all genes upregulated in the “PDL1-up” group) and the metagene PDL1-not-up” (mean expression of all genes upregulated in the “PDL1-not-up” group). This score was then compared between the “PDL1-up” and “PDL1-not-up samples.

### Statistical analysis

Correlations between tumor groups and clinicopathological features were analyzed using the t-test or the Fisher's exact test (variables with 2 groups) when appropriate. Disease-free survival (DFS) was calculated from the date of diagnosis until the date of distant relapse or death from pancreatic cancer. Overall survival (OS) was calculated from the date of diagnosis to the date of death from pancreatic cancer. Follow-up was measured from the date of diagnosis to the date of last news for event-free patients. Survivals were calculated using the Kaplan-Meier method and curves were compared with the log-rank test. Univariate and multivariate survival analyses were done using Cox regression analysis (Wald test). Variables tested in univariate analyses included patients' age at time of diagnosis (continuous value), sex, pathological features including pathological type, tumor size (T2 and T3 *vs* T1), regional lymph node status (positive *vs* negative), tumor grade (2 and 3 *vs* 1), and *PDL1* expression (“up” *vs* “not-up”). Variables with a *p*-value < 0.10 were tested in multivariate analysis. All statistical tests were two-sided at the 5% level of significance. Statistical analysis was done using the survival package (version 2.30) in the R software (version 2.9.1;. We followed the reporting REcommendations for tumor MARKer prognostic studies (REMARK criteria) [81].

## SUPPLEMENTARY MATERIALS FIGURES AND TABLES


















