# Supplementary material for: Prognostic value of PDL1 expression in pancreatic cancer
Source: Oncotarget. 2016 Aug 29;7(44):71198–210. doi: 10.18632/oncotarget.11685 (PMC5342072; doi:10.18632/oncotarget.11685)
Supplement: Supplementary file 1 [file oncotarget-07-71198-s001.pdf]

Prognostic value of *PDL1* expression in pancreatic cancer

Supplementary Material

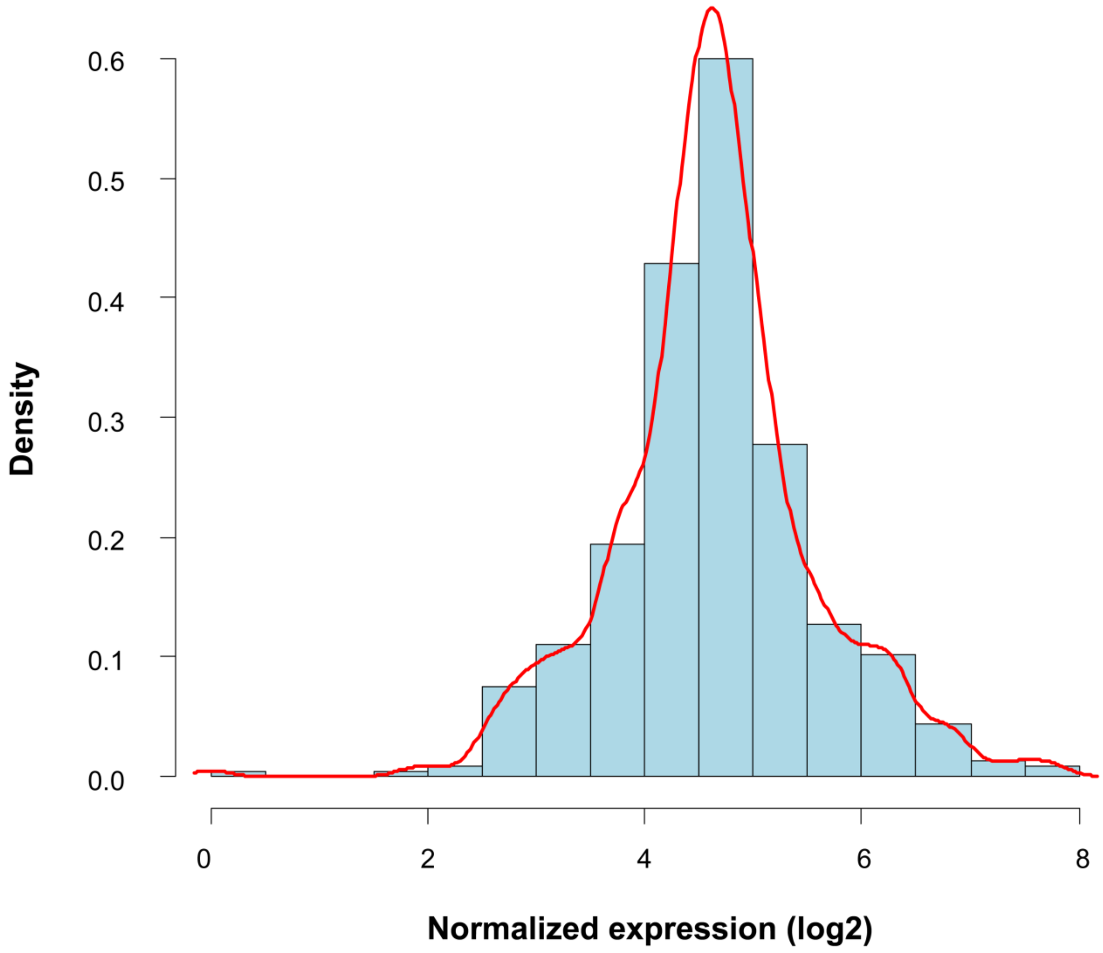

Supplementary Figure 1: Distribution of *PDL1* expression levels across the 453 samples

Normalised data have been log2-transformed. The red line of the histogram represents the density curve of distribution.

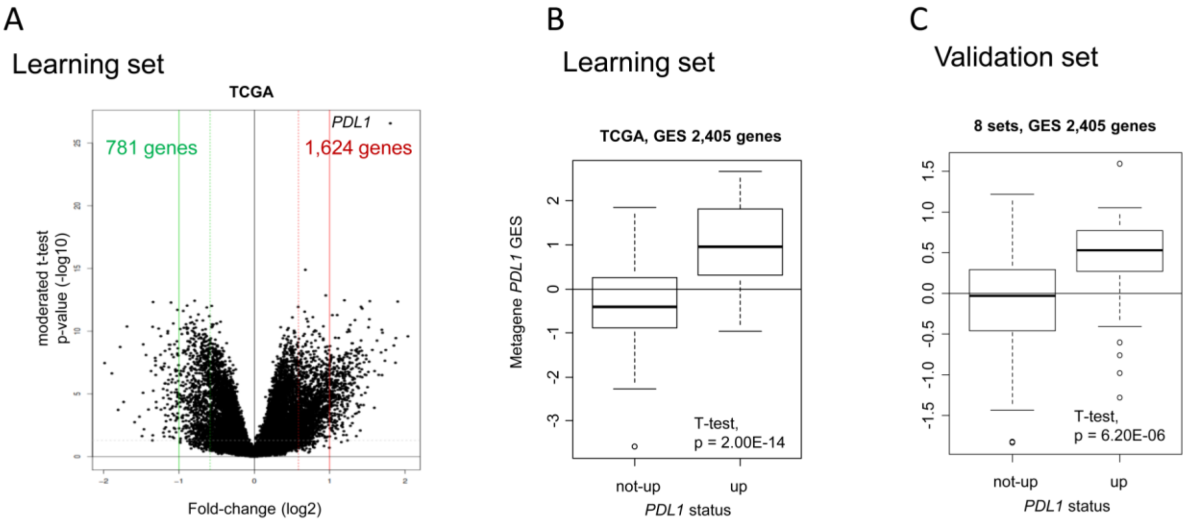

Supplementary Figure 2: Supervised analysis of gene expression profiles between the "PDL1-up" and "PDL1-not-up" sample groups

A/ Volcano plot showing the 2405 genes differentially expressed in the learning set (TCGA). B-C/ The metagene-based prediction score is significantly higher (Student t-test) in the “PDL1-up” samples than in the “PDL1-not-up” samples in the learning set as expected (B), but also in the independent validation set (C).

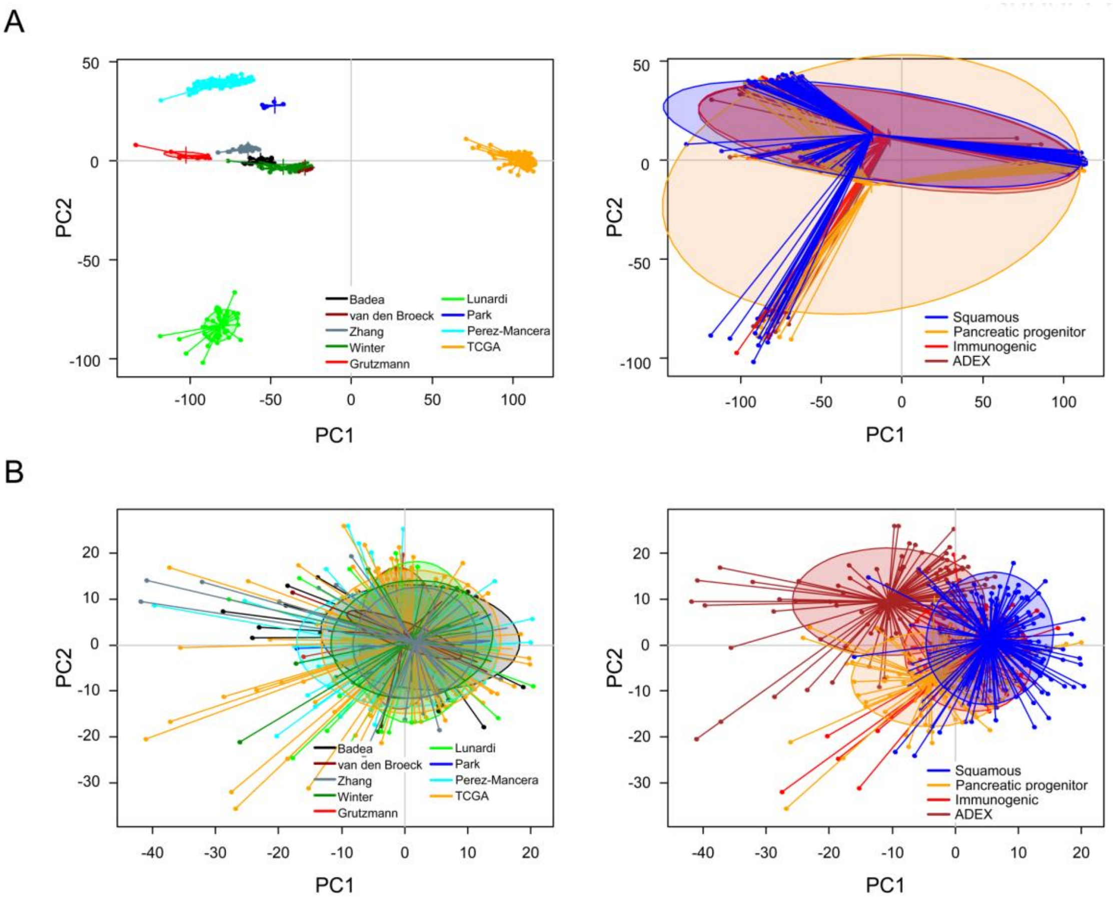

Supplementary Figure 3: Principal component analysis before and after normalization of the nine data sets.

PCA was applied to the 453 cancer samples and the top 2,000 most variable genes extracted from the nine data sets. A/ Before normalization, cancer samples in the 2D scatter plot representation are grouped according to their origin data set (*left*: each color represents a set) and not according to the four Bailey's molecular subtypes (*right*: each color represents a subtype). B/ After normalization, samples are correctly clustered according to their molecular subtypes (*right*), rather than their origin data set (*left*), clearly suggesting that the normalization has removed technical differences in gene expression while maintaining the information relevant to biological differences.

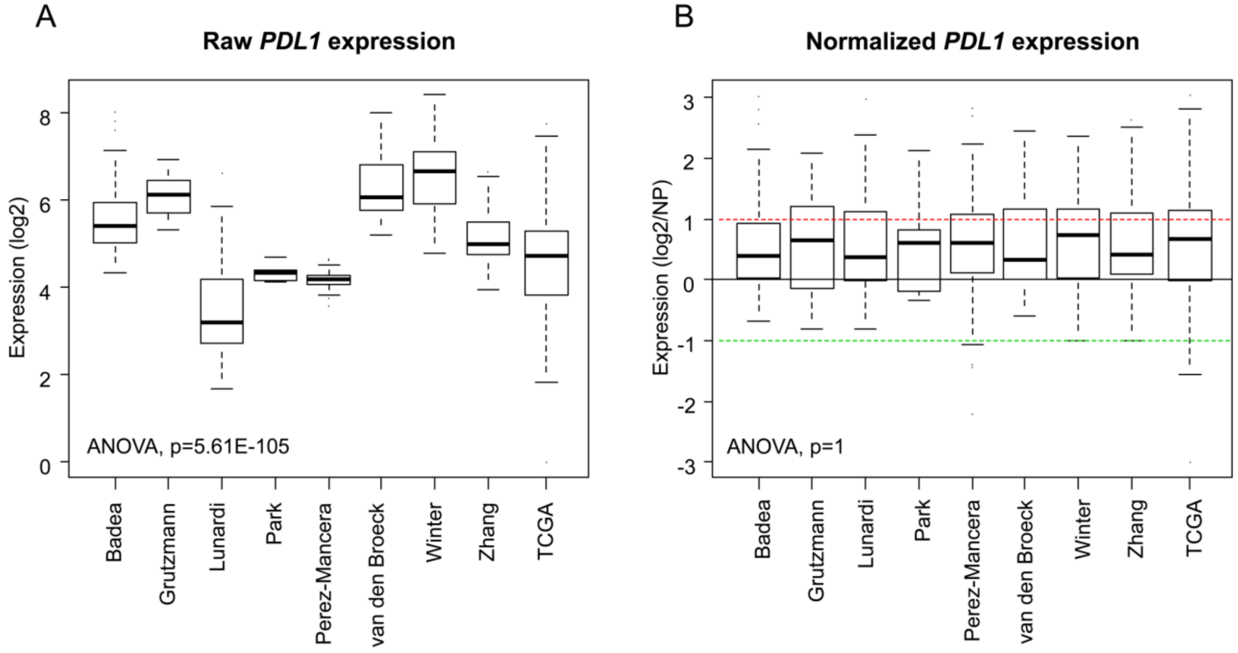

Supplementary Figure 4: Distribution of *PDL1* expression in the nine datasets, before and after normalization.

A/ Before normalization, the distribution is heterogeneous and significantly different between all data sets. B/ After normalization, the distribution is homogeneous and not different between data sets.

Supplementary Table 1: Clinicopathological characteristics of population

Supplementary Table 2: List of 2,405 genes differentially expressed between the "PDL1-up" and "PDL1-not-up" sample groups

Supplementary Table 3: Ontology analysis of the 2,405 genes differentially expressed between the "PDL1-up" and "PDL1-not-up" sample groups

Supplementary Table 4: Correlations between the *PDL1* expression-based groups and immunity-related features in pancreatic cancer, and comparison with breast cancer and GIST

Supplementary Table 5: List of genes differentially expressed between the "PDL1-up" and "PDL1-not-up" sample groups in pancreas cancers (present study), breast cancers (Sabatier et al Oncotarget 2015) , and GISTs (Bertucci et al, Oncoimmunology 2015) generated from the 12,091 genes common to three studies and using the same parameters for supervised analysis (moderated t-test, p<5%, q<25%, |FC|>1.5x)

Supplementary Table 6: Ontology analysis of the genes upregulated in the "PDL1-up" versus "PDL1-not-up" sample groups in pancreas cancers (present study), breast cancers (Sabatier et al, Oncotarget 2015), and GISTs (Bertucci et al, Oncoimmunology 2015)

Supplementary Table 7: List of pancreatic cancer data sets included in the analysis

Supplementary Table 8: List of *PDL1* probe sets analyzed in the DNA microarray-based data sets
